# Diaspora engagement: a scoping review of diaspora involvement with strengthening health systems of their origin country

**DOI:** 10.1080/16549716.2021.2009165

**Published:** 2021-12-14

**Authors:** Editt N. Taslakian, Kent Garber, Shant Shekherdimian

**Affiliations:** a Division of Plastic Surgery, Department of Surgery, University of Washington Medical Center, Seattle, WA, USA; b Department of Surgery, David Geffen School of Medicine, University of California Los Angeles, Los Angeles, CA, USA; c Division of Pediatric Surgery, Department of Surgery, David Geffen School of Medicine, University of California Los Angeles, Los Angeles, CA, USA

**Keywords:** Health care organizations, international health, health systems, diaspora

## Abstract

**Background:**

Diaspora communities are a growing source of external aid and resources to address unmet needs of health systems of their homelands. Although numerous articles have been published, these endeavors as a whole have not been systematically assessed.

**Objective:**

Examine the available literature to assess activities through which diasporas engage with the health system in their origin country and what barriers they face in their interventions.

**Methods:**

This is a scoping review from 1990–2018 using the PRISMA-Scr framework to examine both peer-reviewed and gray literature on (1) specific activities through which diasporas contribute to the health system in their origin country; (2) major health needs diasporas have tried to address; and (3) barriers faced by diaspora healthcare efforts.

**Results:**

The initial search identified 119 articles, of which 45 were eligible after excluding non-relevant studies. These were case studies of diaspora contributions to health systems in their origin country (13), interviews (13), literature reviews (9), general articles on the topic (4), and correspondences or presentations (6). The healthcare needs diasporas have sought to address include health workforce emigration (‘brain drain’) (10), capacity building for research and training (10), inadequate infrastructure (5), and finances (4). Specific activities included short-term missions (11), establishing partnerships (9), emigration back to country of origin (8), specific research and training programs (8), and financial remittances (5). Specific barriers identified were most commonly financial need within the origin country (8), lack of sustainability (6), communication issues (6), lack of intention to return to the origin country (5), infrastructure (4), and political concerns (3).

**Conclusion:**

Further research on how to expand the scope of and reduce barriers to diaspora engagement is needed to optimize the effectiveness of diaspora contributions to their origin countries. Metrics and standards should be developed for assessing impact of diaspora engagement and interventions.

## Background

Diaspora communities refer to populations from within a given territorial, national, or ethnic origin living abroad and having ties and exchanges of various types with the communities both in the origin country and destination country [1]. Over the past quarter-century, diaspora populations have grown significantly, reflecting the unprecedented levels of migration and forced displacement around the world [2].

Diaspora communities can be a powerful source of external development aid to their origin countries. Their contributions take many forms, including remittances (money transfers between individuals), charitable donations, partnerships, skill transfers, policy advising, and other forms of assistance [3]. In 2018, remittance flows of general aid to low and middle-income countries (LMICs) reached a record-high of $529 billion and are projected to become the leading source of external development aid globally within a few years [4,5]. Moreover, diaspora communities in many cases can relate more easily to the culture and practices of the homeland populations and thus can develop long-lasting and effective collaborations [6,7]. Over the past several years, the contributions of diaspora communities to the sustainable development in their origin countries have been acknowledged by global initiatives, including the 2030 Agenda for Sustainable Development, the New York Declaration for Refugees and Migrants, and the Summits of the Global Forum on Migration and Development [8]. Major development agencies, bilateral aid organizations, and various NGOs have commissioned numerous studies and initiatives seeking to better harness diaspora development potential for economic growth [9]. Meanwhile, the growth of social media and other information technologies have helped diasporas organize and increase their visibility and discourse with their home communities [10].

In the health sector, although some articles regarding individual diaspora contributions have been published, these articles have not been systematically assessed. Household surveys in LMICs have demonstrated that a small but substantial percentage of remittances are spent on healthcare needs [11], and diaspora contributions to emergency humanitarian disaster responses are well-known [12]. However, diaspora contributions to healthcare appear to extend well beyond direct payments to individuals and disaster relief. A recent inventory of medical diaspora organizations based in four high-income countries found 89 such groups focusing on health service provisions and training in origin countries, professional networking, and supporting healthcare for displaced persons in host countries. Some of their activities focused on short-term humanitarian needs, but others had longer time frames and health system priorities. That study also found 68 LMICs that had set up diaspora offices to help coordinate or manage diaspora contributions [13].

These findings point to the growing interest from both diaspora organizations and policymakers to capitalize upon and streamline diaspora resources. However, no formal analysis of the priority, scope, and barriers relating to these efforts, based upon published literature, exists. As described by Aguinas, the effectiveness and impact of diaspora-centered institutions are historically difficult to assess; evaluations rarely exist, nor are they publicly available [14]. However, an analysis of the existing literature could provide insight into what is known about healthcare-related diaspora efforts thus far, and identify challenges and opportunities for supporting such efforts in the future. This scoping review seeks to examine the available literature regarding the following: (1) the specific activities through which diaspora communities contribute to the health system of their origin country; (2) the major health needs identified as problems that diasporas have tried to address; and (3) the barriers commonly faced by diaspora healthcare efforts in their countries of origin.

## Methods

This study was designed as a scoping review using the Preferred Reporting Items for Systematic Reviews and Meta-Analysis Extension for Scoping Reviews (PRISMA-ScR) checklist. Our goals were to map the available literature regarding diaspora contributions to the health system in their origin countries and the specific activities performed, needs identified, and barriers encountered in these efforts. As the literature on this topic is diverse, from peer-reviewed articles to gray literature, we cannot compare articles to one another as would be done in a systematic review. However, we can identify the types of available evidence on this subject.

We conducted a systematic search of the literature published in the English language from 1990–2018 to capture diaspora activities over the past quarter-century. The databases searched for peer-reviewed publications included PubMed, Google Scholar, Ovid MEDLINE, Ovid Embase, Business Source Premiere, and ERIC databases. The initial search terms were as follows: (‘diaspora’) AND (‘health’ or ‘healthcare’ or ‘medicine’ or ‘health system’) AND (‘contribution’ or ‘assistance’ or ‘aid’ or ‘training’). Search terms were entered as specific keywords rather than Medical Subject Heading (MeSH) terms as the latter produced an unacceptably broad set of search returns that were not specific to diaspora-related activities. Additionally, the gray literature was searched for non-peer-reviewed articles, using the same terms as above as well as additional terms sometimes used to describe characteristics of diaspora communities, including ‘expatriate,’ ‘migrant,’ ‘foreign-born,’ and ‘returnee.’ This search included archives of major development agencies such as the World Bank, World Health Organization, U.S. Agency for International Development, the Department for International Development, Deutsche Gesellschaft fur Internationale Zisammenarbeit (GIZ) GmbH, Asian Development Bank, and Migration Policy Institute. Any additional citations from the supplemental review of reference lists from the search were also screened by the authors.

Articles were excluded if they were published prior to 1990, or if upon reading the abstract or text they were found to be not directly related to diaspora contributions to the health system of their origin countries. For example, articles regarding medical tourism, non-health related diaspora contributions to the country of origin (for example business-related, financial, other skills development, etc.), or health workforce emigration (also known as ‘brain drain’) without the involvement of diaspora activities were excluded.

Data was extracted with the following elements: author, publication year, type of study or article, diaspora origin country, diaspora destination country, the targeted need of origin country, type of intervention, evidence of impact (if any), evidence of sustainability (if any), and challenges or barriers identified to the activity. Data were charted independently by two authors (ET, KG) using a pre-developed spreadsheet containing separate columns for the above-specified elements. Results were compared and differences in data extraction or interpretation were discussed and, when necessary, reconciled by the third author (SS).

## Results

**Figure 1.**
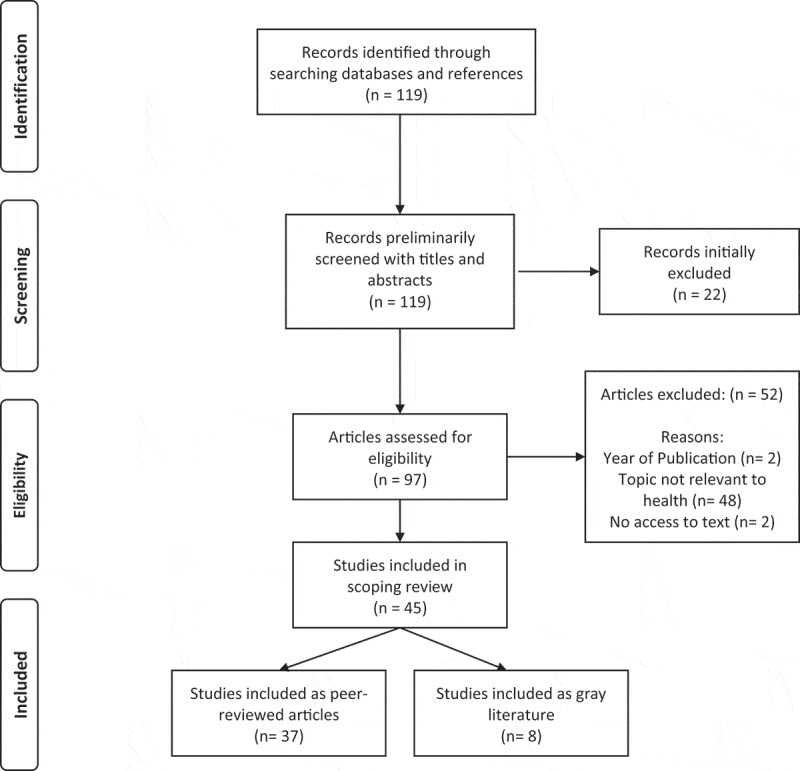
PRISMA flow diagram of scoping review articles [60]

The search of peer-reviewed and gray literature available initially yielded one hundred and nineteen articles. Of these, ninety-seven were selected for full-text review after preliminary screening of titles and abstracts. Following the review, forty-five were eligible for the purposes of this scoping review (Figure 1).

In terms of article types, thirteen were case studies of specific diaspora contributions to health systems in their origin countries [15–27]; thirteen were studies involving interviews of health workers and key stakeholders [28–40]; nine were literature or systematic reviews of specific topics within the realm of diaspora healthcare aid to the origin country, for example HIV/AIDS financing in sub-Saharan Africa, disease control efforts, health system reviews of the origin country including aid from the diaspora, and reviews of institutions maintaining relationships with diaspora communities [12,14,41–47]; four were general articles on the topic [48–51]; and six were miscellaneous articles or correspondences regarding the topic [6,52–56].

Geographically, seventeen articles focused on diaspora contributions in sub-Saharan African countries, twelve in Eurasian countries, six in the Middle East/North Africa region, two in Southeast Asia, and six articles were global in scope or covered multiple regions (see Table 1). Specific countries with the highest number of articles were South Africa, India, Nepal, Somalia, Zimbabwe, and Ethiopia. No studies were found from Central or South American geographical regions in the English language. Most of the articles (84%) were written in the last 10 years.

**Table 1.** Geographic distribution of studies on diaspora healthcare contributions

Country	Number of articles	Region	Number of articles
Mixed Countries/General	14	Sub-Saharan Africa	17
South Africa	3	Eurasia	12
Somalia	2	Middle East/North Africa	6
Nepal	2	Southeast Asia	2
India	2	Mixed/General	8
Zimbabwe	2		
Ethiopia	2		
Liberia	1		
Armenia	1		
Sudan	1		
Nigeria	1		
Cape Verde	1		
Israel	1		
Albania	1		
China	1		
Bangladesh	1		
Senegal	1		
Ghana	1		
Korea	1		
Indonesia	1		
Philippines	1		
Malawi	1		
Pakistan	1		
Rwanda	1		
Botswana	1		

Of the healthcare needs diaspora organizations have sought to address, health workforce emigration (ten articles) and limited capacity for medical research and quality training (ten articles) were most commonly discussed, followed by inadequate infrastructure (five articles) and finances (four articles) for various disease control efforts and quality improvements (Table 2). Discussions of reversing health workforce emigration, also known as brain drain, tended to focus on the need for better quality in physician and healthcare training, rather than recruitment of any specialty-specific physicians, although one article discussed the lack of quality surgery-specific care [6]. Of the articles that focused on capacity building for research and quality, the most commonly identified needs were lack of international collaborations and networking opportunities, disease screening and primary prevention (such as for HIV), and lack of academic career development opportunities in national universities. Infrastructure problems that were cited included understaffed facilities, lack of financial commitments by the state to complete large projects, lack of nutrition resources (regarding maternal and child health in particular), and lack of data to set priorities [56]. For example, Poppe et al. conducted interviews with 27 sub-Saharan African health workers in Belgium and Australia and found that institutional and structural crises in their source countries, in combination with the positive living conditions in Europe, played a major role in their lack of intention to return, including concerns over health system infrastructure as well as the quality of education and security [30]. Fourteen articles discussed specific mechanisms or processes by which needs were identified, including surveys of expatriates and key stakeholders, formal needs assessments, and interviews of key informants.

**Table 2.** Needs identified, activities undertaken, and barriers encountered by diaspora medical organizations

Needs	Activities	Challenges
Brain drain/lack of appropriately trained medical professionals	Short-term mission trips and aid	Extent of financial need of home country
Poor capacity for research and quality training	Establishing partnerships	Lack of sustainable follow through
Inadequate health system infrastructure	Return emigration	Lack of structured and regular communication
Limited health financing	Improving research and training programs	Lack of intention to return to origin country
	Financial remittances	Poor infrastructure
		Political instability

Specific activities undertaken by diaspora organizations were varied, including short-term mission trips and humanitarian or disaster aid (eleven articles) [12,17,23,24,29,30,39,40,42,49,54], establishing partnerships for knowledge and skill transfer with the diaspora (nine articles) [14,15,18,31,34,45,46,50,52], emigration back to country of origin (eight articles) [16,19,21,28,30,32,33,47], improving research and training programs within the country itself (eight articles) [18,20,22,25,27,48,51,53], and providing financial remittances (five articles) [21,41,43,44,55] to the country of origin (Table 2). For studies that cited partnerships or training programs, these efforts commonly occurred between diaspora organizations and local health facilities or academic institutions. Several articles cited the UN Transfer of Knowledge through Expatriate Nationals (TOKTEN) program, which started in the late 1970s to recruit diaspora individuals to volunteer for short periods of time in their origin countries to provide mentoring or technical advising to the professionals in the origin country. Only one study cited unique partnerships or relationships between the diaspora organization and a government entity such as the ministry of health [14], including examples from Haiti, Bangladesh, Benin, Dominica, Georgia, India, Armenia, Lebanon, Mali, Serbia, Somalia, Sri Lanka, Tunisia, Yemen, and Syria within which there is a focus from government ministries to develop stronger links with the diaspora. Of those studies reporting return-country emigration, factors appearing to influence such decisions included poor work environment, insufficient pay, unsecured accommodation, political instability, poor education for children, and fear for personal safety.

Many barriers to effective diaspora engagement were cited, most commonly financial need of the origin country (eight articles) [12,28,30,32,40,41,44,51], followed by lack of sustainable follow-through by either the programs or the origin country (six articles) [19,23,37,39,48,56], lack of structured and regular communication among the partner organizations and the origin country (six articles) [15,16,27,31,34,56], lack of general intention to return to the origin country (five articles) [21,26,30,31,35], poor infrastructure (four articles) [17,37,40,48], and concerns of political instability (three articles) [30,36,51] (Table 2). When authors cited ‘financial need of the origin country’ as a barrier, they typically meant that diaspora organization efforts were too small or under-resourced to effectively impact the scale of the identified problem. Reasons for poor follow-through and lack of sustainability included lack of commitment from government agencies [12], lack of overall resources [15,48], lack of appropriate organization and ongoing supervision of interventions [40], lack of indicators to regularly assess levels of engagement [39], and lack of structured communication between partners [15]. The most commonly suggested option for overcoming these barriers included increasing levels of support for diaspora activities by government agencies and local partners. Of the studies included in this review, none had specific objective metrics for measuring the effectiveness of interventions or activities to overcome barriers.

## Discussion

To our knowledge, this is the first study to review the existing literature on the topic of diaspora contributions to the health systems of their origin countries. There are inherent limitations to this study, as scoping reviews are intended to map the available literature rather than to provide higher quality assessment or extensive data analysis of the content found [57]. There is an overall lack of available literature published since 1990 regarding the topic of diaspora contributions to the health systems of their countries of origin. Much of the literature cited in this study lack objective measures of the outcomes of the programs or efforts described. In this regard, these studies cannot be compared side-by-side as they differ greatly in style and scope. There is ample opportunity to improve the available evidence base of these efforts should diaspora organizations choose to publish high-quality, peer-reviewed literature regarding their efforts and outcomes.

Limited as they may be, the overall findings of this scoping review suggest that diaspora communities have most frequently sought to address health workforce emigration and capacity-building needs for research and training for quality improvement. To address health workforce emigration, suggestions have included short-term return and facilitation of more permanent return through stakeholder collaboration [40], as well as scholarship schemes for training new doctors and nurses [17], telemedicine, and educational webinars [50]. The literature suggests that the most common activities that diaspora organizations have used to address the needs of their countries of origin have been through short-term medical missions and partnerships with the government or local organizations. However, long-term success in the sustainability of such programs has been hindered by financial issues within the origin country and lack of structured communication and follow-through, as well as a lack of general intention to return to the origin country, poor infrastructure, and political instability. One partnership that has shown success against this barrier is the Alumni Diaspora Fellowship Program between the University of Witwatersrand (South Africa) and Vanderbilt University Medical Center (USA) as described by Kramer and Zent in 2019, serving as a more recent example of a collaboration that is well-funded, allowing both the diaspora alumni to return to their country of origin for the partnership and also fund Wits researchers to visit the alumni in the USA, creating a two-way interaction and knowledge exchange [7].

Importantly, only a handful of the studies make explicit mention of how intervention priorities were selected. In the development sector, large organizations typically conduct formal needs assessments, stakeholder interviews, or surveys to identify priorities for investment or assistance, as well as to identify actors in the health space (e.g. NGOs, government, development agencies) that may be doing similar work. These types of formal assessments help ensure that interventions are appropriately tailored to local needs, avoid duplicating work that others may already be doing, prevent parallel systems from being set up, and establish a baseline against which impact can be assessed. From the studies reviewed in this analysis, it does not appear that these types of formal assessments are routinely done by diaspora health organizations, or at least they are not published in the literature if they do. This raises a critical challenge for optimizing diaspora contributions.

Several of the studies reviewed offered suggestions for how diaspora contributions might be more effectively leveraged, and how existing barriers could be overcome. These suggestions included encouraging diaspora healthcare professionals to return (short- or long-term) and teach at academic centers in the origin country as a means of ‘brain exchange,’ government recognition and support of medical diaspora organization activities, pooled funding models among organizations for larger investments, the establishment of Ministry-level institutions for diaspora engagement, telemedicine, and more.

It is evident that some barriers are more easily addressed than others. For example, government corruption has been cited as a deterrent to investment for certain diaspora communities. In those contexts, diasporas might be inclined to seek sustainable partnerships with more trustworthy partners, such as local academic or clinical institutions. At the same time, more needs to be understood about the effectiveness of government diaspora agencies in engaging diaspora stakeholders in policy discussions. These offices, which now exist in dozens of LMICs, could theoretically help to harmonize the efforts of diaspora organizations as well as other influential stakeholders, including development banks, NGOs, etc., towards tackling major problems within the origin country’s health system. However, little is known about what these offices have achieved, and how diaspora organizations view and interact with them. Ahmed et al. describe how in the current era of globalization, governments must better capitalize on their diaspora as an underutilized resource to reduce inequalities in care in their countries of origin [6]. Anand et al. in 2009 describe how diaspora scientists in the USA feel personally accountable to both the U.S. and their origin country institutions, which can help with research and training collaborations among the two for capacity building [48]. Frehywot et al. in 2019 published an inventory of low- and middle-income countries’ medical diaspora organizations from the USA, the UK, Canada, and Australia, showing a trend in three focuses of these organizations: providing healthcare services, training, and humanitarian aid when needed to their origin country; creating professional networks of migrant physicians; and supplying improved and culturally sensitive healthcare to the migrant population within the host country [13]. The lack of available data on diaspora organizations, however, remains a limitation in the identification and analysis of these efforts. There is an overall scarcity of articles available in the literature to discuss the role of diasporas and their activities. Similarly, there is a lack of guidance or standards for guiding diaspora engagement, such as a quality checklist for needs assessments, interviews, and the like in the initial determination of diaspora activities. There are toolkits to help start research partnerships, such as from the Canadian Coalition of Global Health Research (CCGHR) Principles for Global Health Research [58] and the UK Collaborative on Development Research (UKCDR) [59, 60] available online, however, there are currently no formal guidelines specific for diaspora organizations to use to develop and measure the effectiveness of contributions to their origin countries. Such resources are paramount to the successful and meaningful bridging of diaspora aid and origin country needs. Government agencies involved with diaspora engagement can spearhead needs assessments of the healthcare sector within their countries by interviewing key stakeholders and organizations and then communicating these perceived needs to diaspora organizations that are interested in providing aid. Further assessments of how to expand the scope of and reduce barriers to diaspora engagement are also needed to optimize the effectiveness of diaspora contributions to their origin countries.

The establishment of a set of basic tools and indicators would be of great value for guiding diaspora activities and assessing the impact of diaspora interventions. For example, surveys could be created and validated to assess the diaspora perception of efforts and allow comparisons to the origin country organizations’ perceptions of the same efforts; these activities could help assess outcomes and barriers and guide future directions for ongoing partnership work. It is also important to note that engaging diaspora communities may not always have a positive outcome, and literature about failed partnerships or negative viewpoints regarding diaspora engagement may be rare. For example, Parekh et al’s article from 2016 discusses Malawian impressions of expatriate physicians as negative due to lack of understanding of culture-specific issues, poor adaptation to low resource settings, communication issues with patients, and self-serving or exploitative intentions [31]. This was the only study in our scoping review that directly focused on this topic. More formal assessments of the perceptions towards and impact of diaspora projects by the local communities they affect are needed to objectively characterize the impact.

Limitations of this study include inherent barriers with scoping reviews, such as a broad initial search with multiple structured iterations and hand-searching the literature. Inconsistencies are possible in the generalizations and grouping of articles as there was no consistent metric common among the articles for comparison. Many of the articles were found in the gray literature, which may be a weaker source of evidence compared to peer-reviewed articles. Our search included only English-language papers and thus we did not capture articles in other languages that might otherwise contribute to this review. Furthermore, our study was not designed to capture the work of diaspora individuals or groups involved with their origin countries who did not explicitly mention in their publications that they were members of a diaspora community. Our results and conclusions may be biased by the fact that much of the work done by diasporas is not published and is therefore under-reported, particularly if the efforts are not considered a success and not documented in the literature. As our study is based on published reports, we are likely capturing only the tip of the iceberg regarding diaspora contributions to health systems in their origin country, and there is an overall lack of metrics or other data to compare or track outcomes over time in a standard fashion.

## Conclusions

The available literature suggests the most prominent areas of healthcare engagement by diaspora communities with their origin countries include addressing health workforce emigration and building capacity for research and training. Diaspora contributions to healthcare in their origin countries have largely focused on short-term missions and developing partnerships, however, sustainability of the programs and finances have been a common barrier to successful long-term engagement. More attention should be given toward sustainable responses that integrate into the origin country’s health system, such as performing needs assessments for better preparation and allocation of resources. In addition, metrics and standards should be developed for assessing the impact of diaspora engagement and interventions, as, despite many ongoing organizations and activities worldwide, such resources do not exist. As the current literature on the topic of diaspora engagement with the health system of their country of origin is limited, further and more formal assessments of these activities will be paramount to understanding and ultimately optimizing the efforts of diasporas in improving the quality of the health system in their origin countries.

## Paper context

The efforts of diasporas as a growing source of aid to address the needs of healthcare systems of their origin countries have not been systematically assessed. Our scoping review examines the literature about how diasporas contribute to healthcare in their home country and the barriers faced. Further research on how to expand the scope of and reduce barriers to diaspora engagement is needed, and standards should be developed for assessing the impact of diaspora interventions.
